# A Virtual In-Cylinder Pressure Sensor Based on EKF and Frequency-Amplitude-Modulation Fourier-Series Method

**DOI:** 10.3390/s19143122

**Published:** 2019-07-15

**Authors:** Qiming Wang, Tao Sun, Zhichao Lyu, Dawei Gao

**Affiliations:** 1 School of Mechanical Engineering, University of Shanghai for Science and Technology, Shanghai 200093, China; 2 School of Automotive Studies, Tongji University, Shanghai 201804, China

**Keywords:** in-cylinder pressure identification, speed iteration model, EKF, frequency modulation, amplitude modulation

## Abstract

As a crucial and critical factor in monitoring the internal state of an engine, cylinder pressure is mainly used to monitor the burning efficiency, to detect engine faults, and to compute engine dynamics. Although the intrusive type cylinder pressure sensor has been greatly improved, it has been criticized by researchers for high cost, low reliability and short life due to severe working environments. Therefore, aimed at low-cost, real-time, non-invasive, and high-accuracy, this paper presents the cylinder pressure identification method also called a virtual cylinder pressure sensor, involving Frequency-Amplitude Modulated Fourier Series (FAMFS) and Extended-Kalman-Filter-optimized (EKF) engine model. This paper establishes an iterative speed model based on burning theory and Law of energy Conservation. Efficiency coefficient is used to represent operating state of engine from fuel to motion. The iterative speed model associated with the throttle opening value and the crankshaft load. The EKF is used to estimate the optimal output of this iteration model. The optimal output of the speed iteration model is utilized to separately compute the frequency and amplitude of the cylinder pressure cycle-to-cycle. A standard engine’s working cycle, identified by the 24th order Fourier series, is determined. Using frequency and amplitude obtained from the iteration model to modulate the Fourier series yields a complete pressure model. A commercial engine (EA211) provided by the China FAW Group corporate R&D center is used to verify the method. Test results show that this novel method possesses high accuracy and real-time capability, with an error percentage for speed below 9.6% and the cumulative error percentage of cylinder pressure less than 1.8% when A/F Ratio coefficient is setup at 0.85. Error percentage for speed below 1.7% and the cumulative error percentage of cylinder pressure no more than 1.4% when A/F Ratio coefficient is setup at 0.95. Thus, the novel method’s accuracy and feasibility are verified.

## 1. Introduction

The development of cleaner and more efficient engines requires the continuous measurement of in-cylinder pressure which is of fundamental importance for combustion optimization, air-fuel ratio control, noise and pollutant reduction. More specifically, cylinder pressure is a fundamental intermediate variable that indicates engine state and drives models for engine combustion control. High-bandwidth control of ignition and the air-fuel ratio may permit these engines to operate reliably near the lean-burn limit, with significant improvements in efficiency and decreased emission of CO and NOx. Fully utilizing this technology requires a key factor: real-time cylinder pressure [1]. The goal of Rizzoni’s [2] method is to build a stochastic model for the combustion pressure process in the spark-ignition engine. Control of torque balance can improve drivability and can suppress noise from light-duty diesel engines, but as a crucial part of the control algorithm, obtaining pressure data necessitates expensive pressure sensors and demands considerable computational time [3]. The advent of cylinder-pressure transducers seems promising, boasting a better approach to detect the in-cylinder pressure; however, its reliability, complex installation, high cost, and short working lifespan are full of controversy. As new technologies improve, such as computational methods, reconstructing cylinder pressure in a multi-cylinder internal combustion (IC) engines becomes more feasible. Bennett [4] proposed a robust adaptive-gradient descent-trained NARX neural network using both crank velocity and crank acceleration as inputs to reconstruct cylinder pressure in multi-cylinder IC engines. Another work has shown comprehensive results from an experimental assessment of a common rail diesel engine operated with neat diesel fuel and with ethanol substitutions [5]. With different substitutions and different substitution rates, pressure varies rapidly. Researchers have pursued other various approaches to develop a comparatively optimal way to obtain the desired cylinder pressure, directly or indirectly. Rizvi [6] proposed a novel method to detect engine misfire faults based on a hybrid model of the gasoline engine. As this method mainly employs cylinder pressure, closed-loop [7] combustion control [8] becomes feasible and misfire can be detected in multiple ways. However, these approaches vary in cost, reliability, robustness, accuracy, and convenience. Therefore, a low-cost [9] noninvasive soft-pressure sensor with a high level of reliability and accuracy is necessary. Payri [10] presented a step-by-step approach to optimize the signal processing both for offline and online applications based on the characteristics of the signal. Maurya [11] proposed that a method based on standard deviation of pressure, and pressure rise rate is used to find the minimum number of engine cycles to be recorded for averaging to get reasonably accurate pressure data independent of cyclic variability. Bhatti [12] proposed that two robust second-order sliding mode observers are employed that require two state mean value engine models based on inlet manifold pressure and rotational speed dynamics. The design of the second-order sliding mode observer depends largely on the model accuracy. And, more importantly, this method had the problems of chattering and large initial error. As a result, the tracking error of the steady-state speed was only about 4% and required a certain convergence time. Ferrari [13] proposed that pressure of nozzle opening and closing was identified by means of pressure data fitted by bench test. Then the time-frequency analysis was used to obtain the mean instantaneous frequency and adjust the control strategy of the injector. However, this method did not process data effectively, such as filter, before fast Fourier time-frequency transform, so this may affect the accuracy. Eriksson [14] calculated the corresponding relationship between pressure value and crankshaft rotation angle. The error of peak identification based on ion current in (Eriksson, 2003, Figure 4) was still large. Comparing with the corresponding relationship proposed by Eriksson [14], the cylinder pressure time curve is presented in this paper. It is based on burning theory and Law of energy conservation, and then modified by Extended-Kalman-Filter-optimized (EKF) engine model, which has a higher stability.

Observer-based method [12,13,14,15] is proven to be a good way to predict the output of SISO system. Engine cylinder pressure signal can be used to characterize the engine combustion state [16,17]. Due to the influence of multiple variables, it is difficult to establish a precise physical model of cylinder pressure. However, by observing and analyzing the cylinder pressure signal, this signal is cyclical with variable frequency (associated with speed) and variable amplitude (associated with the peak combustion pressure). Meanwhile, the engine cylinder pressure signal has a delay characteristic, so it is necessary to predict the current cylinder pressure value by the input of the current moment.

In this paper, a novel method which can identify engine pressure data accurately will provide a spacious viewpoint and means to process signals, thereby achieving important information for practical engineering. This novel method involving Frequency-Amplitude Modulated Fourier Series (FAMFS) and Extended-Kalman-Filter-optimized (EKF) engine model for identifying the periodic signal with its variable frequency and amplitude is proposed. This cylinder pressure identification method is also called a virtual cylinder pressure sensor.

**Figure 1:**
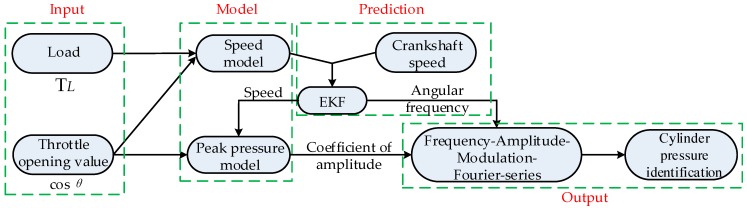
Flow chart of method.

Figure 1 shows a flow chart of method. Crankshaft speed [18,19,20] plays an important role in identifying the pressure as well as other applications, especially in misfire detection [21]. Instantaneous speed fluctuations [22], crankshaft segment acceleration, and transient rotational speed are the most widely utilized variables to locate misfire. Crank speed can also reveal and identify real-time engine combustion parameters [20], which is adopted in this paper. Speed data is affected by measurement noise from the crankshaft and process noise from the engine. These noise sources result in a huge accumulation error during pressure measurements, and EKF is chosen to optimize prediction and filtering [23,24].

The crankshaft speed in one solution step is related to the speed of the last step, so the speed model is an iterative process. Firstly, a novel speed iteration model based on burning theory and Law of energy Conservation is proposed. This iteration model is associated with the throttle opening value and the load. The EKF is then used to estimate the optimal output of the speed iteration model, and this optimal output can then be used for computing the frequency and amplitude of the cylinder pressure cycle-to-cycle. Secondly, taking Otto cycle of standard four-stroke engine as an example, a 24th order Fourier series is chosen to fit the standard working cycle. Thirdly, associated with the variable frequency and amplitude, a frequency-amplitude modulation method is adopted to modulate the standard pressure model identified in step two. Finally, system performance is evaluated by an actual working engine. Therefore, the main contributions of this paper can be summarized as follows:

(1) Aimed to identify in-cylinder pressure and its periodic signals with variable frequency and amplitude, a virtual pressure sensor for engine healthy monitoring with low-cost, real-time, non-intrusive, a long-lifespan, and high-reliability in severe working conditions is presented.
(2) A novel speed model is established according to burning theory, Law of energy Conservation and EKF. The FAMFS method is next developed to fit the periodic signals with variable frequency and variable amplitude.
(3) The proposed method can be applied to multi-cylinder internal combustion (IC) engines including four-cylinder engines.

In this paper, on the one hand, as the key parameter of cylinder pressure identification, the speed iterative model using the EKF has been predicted with high accuracy. However, the overall deviation came from the calculation of pressure due to the unpredictable process of power stroke. On the other hand, due to only related to the crankshaft speed and the amount of air and fuel injection in engine, this method can be applied to multi-cylinder internal combustion (IC) engines including four-cylinder engines. More specifically, many key parameters of ignition engine such as spark advance angle (SAA) need to be considered in the process of building virtual in-cylinder pressure sensor model. Accordingly, at present, the application of the virtual sensor is only limited to spark ignition engines.

## 2. Crankshaft Speed Model

### 2.1. Design of Speed Iteration Model

As generally known, the angular acceleration of crankshaft in one cycle is produced by the output power of the engine, and the output energy per cycle is determined by the amount of air and fuel injected into engine. In internal combustion engines, the amount of air and fuel trapped in the cylinder is relatively influenced by engine speed through the volumetric efficiency, and mainly depends on the engine load. The change of engine load will affect crankshaft speed; hence the amount of air and fuel is deemed relating to the speed. Therefore, it forms a closed-loop iteration process. The crankshaft speed model (CSM) is established in two parts: constant load (Part A) and variable load (Part B). In Part A, the amount of air and fuel is related to the speed of the previous cycle. The power produced through fuel burning compels the crankshaft to rotate; and in Part B, the engine overcomes the load with the price of crankshaft speed reduction. The complete model is then forged by the combination of these two parts.

#### 2.1.1. Constant Load Condition

In the following process, the load is constant. Cylinder pressure is directly related to the amount of fuel and air injection in engine. Thus, the air volume is equal to the engine displacement. The air mass flow of the intake manifold can be calculated as

(1) $${\dot{m}}_{air}=\frac{S_{eff}C_{q}C_{m}P_{im}}{\sqrt{T_{im}}}$$

where, $S_{eff}$ is the active area of throttle valve; $C_{q}$ is the flow coefficient; $C_{m}$ represents the quality factor, associating with the engine. Once the engine model is specified, these parameters such as $S_{eff}$, $C_{q}$ and $C_{m}$ are determined. $T_{im}$ and $P_{im}$ are the temperature and pressure of intake manifold, respectively.

According to the actual working conditions, air mass is then obtained by Refs. [25,26,27,28]:

(2) $$m_{air}=A(\varepsilon )\int_{0}^{t}{\dot{m}}_{air}dt\approx \frac{\pi A(\varepsilon ){\dot{m}}_{air}}{N}=\frac{\pi A(\varepsilon )}{N}\frac{SC_{q}C_{m}P_{im}}{\sqrt{T_{im}}}(1-\cos\theta )$$

where, the value for ε is the engine volumetric coefficient and considered constant when the crankshaft speed does not dramatically change. The integration of air mass flow will inevitably produce a constant term. The sum of constant term and non-constant term is air mass. Since there must be a proportional relationship between the constant term and the non-constant term, in order to facilitate the subsequent calculation, the proportional relationship is defined as A(ε), so it is considered to be the engine parameter coefficient related to ε. Once the engine model is specified, these two parameters are determined. In addition, $t=\frac{\pi }{N}$, and t is opening time of intake valve, and N is the average value of speed in one working cycle.

Different fuels and different fuel ratios will strongly affect the combustion state of the engine [5]. The main elements in the fuel are oxygen, hydrogen, and carbon, with other elements being negligible. Thus, the relationship between mass fractions for the oxygen element $w_{o}$, hydrogen element $w_{h}$, and carbon element $w_{c}$ in 1 kg of fuel are shown below:

(3) $$w_{c}+w_{h}+w_{o}=1$$

The theoretical air volume for 1 kg of fuel that is entirely burned is shown in Equation (4):

(4) $$L_{O}=\frac{22.4}{0.21}(\frac{w_{C}}{12}+\frac{w_{H}}{4}-\frac{w_{O}}{32})$$

The engine output power is determined by the calorific value of the gas mixture, which can be defined as (considering 1 kg of fuel)

(5) $$Q_{mix}=\frac{h_{u}}{22.4\times (\lambda \frac{L_{O}}{22.4}+\frac{1}{M_{\tau }})}$$

where, λ is the excess-air factor; $h_{u}$ is the low calorific value of the fuel; and $M_{\tau }$ is the relative molecular mass of fuel. Therefore, the power output related to the throttle valve is defined as

(6) $$Q=\left[(L_{O}\rho_{air}+1)\frac{m_{air}}{4\rho_{fuel}L_{O}\rho_{air}}\right]\frac{h_{u}}{22.4\times (\lambda \frac{L_{O}}{22.4}+\frac{1}{M_{\tau }})}$$

where, $\rho_{air}$ and $\rho_{fuel}$ is the density of air and fuel, respectively. By substituting Equation (2) into Equation (6), Equation (6) then becomes

(7) $$Q=\left[(L_{O}\rho_{air}+1)\frac{1}{4\rho_{fuel}L_{O}\rho_{air}}\frac{\pi A(\varepsilon )h_{u}}{\left(\lambda L_{O}+\frac{22.4}{M_{\tau }}\right)}\frac{SC_{q}C_{m}P_{im}}{\sqrt{T_{im}}}\right]\cdot \frac{\left(1-\cos\theta \right)}{N}=B\frac{\left(1-\cos\theta \right)}{N}$$

where,

(8) $$B=(L_{O}\rho_{air}+1)\frac{1}{4\rho_{fuel}L_{O}\rho_{air}}\frac{\pi A(\varepsilon )h_{u}}{\left(\lambda L_{O}+\frac{22.4}{M_{\tau }}\right)}\frac{SC_{q}C_{m}P_{im}}{\sqrt{T_{im}}}$$

**Figure 2:**
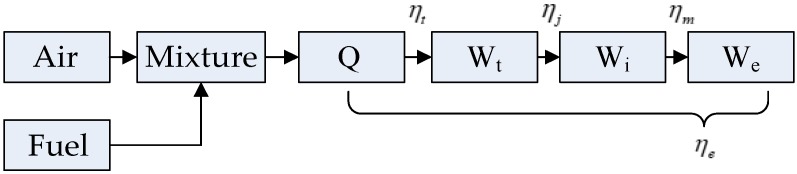
Flowchart of Engine energy transfer.

As widely known, the total energy produced through burning cannot be completely converted to useful work. The process of energy transfer is shown in Figure 2.

$\eta_{t}$ is the theoretical thermal efficiency; $\eta_{j}$ is the loss coefficient; $\eta_{m}$ is the mechanical efficiency; and $\eta_{e}$ is the engine efficiency.

**Figure 3:**
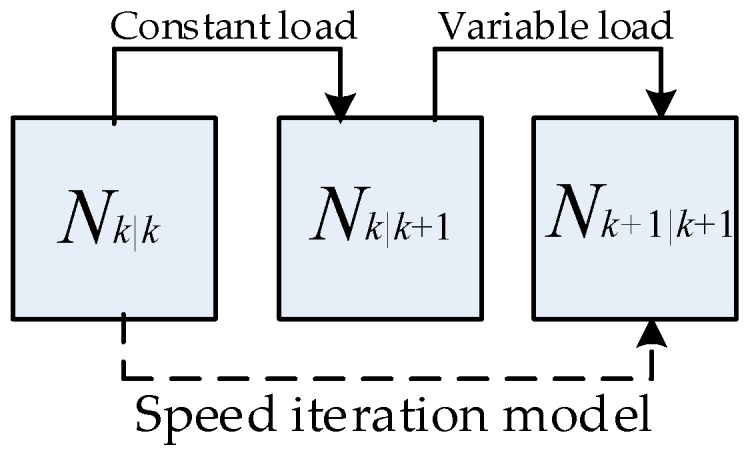
Relationship of speed in the iteration model.

In a four-cylinder four-stroke engine, each cylinder burns once per cycle, and the crankshaft is rotated 720 degrees in one working cycle.

(9) $$W_{e}=U_{kin}(\varphi )+U_{pot}(\varphi )=\frac{J}{2}N^{2}$$

where, $U_{kin}(\varphi )$ is the kinetic energy of the system; $U_{pot}(\varphi )$ is the potential energy of the system; J is the rotational inertia parameter (in this paper, it is equal to a flywheel’s rotational inertia parameter [24]); and φ is the crankshaft angle. By combining Equations (7)–(9), the CSM for Part A is shown in the following equation, and the relationship between $N_{k|k}$ and $N_{k|k+1}$ is shown in Figure 3.

(10) $$\eta_{e}B\frac{\left(1-\cos\theta \right)}{N_{k|k}}=\frac{J}{2}N_{k|k+1}^{2}$$

#### 2.1.2. Variable Load Condition

The change of engine load will affect crankshaft speed. This signal is discretized by computing the mean value of crank speed in power stroke. Since the time step of power stroke is enough small, Equations (11) and (12) are approximately satisfied:

(11) $$T-T_{L}=J\frac{dN}{dt}=J\frac{N_{k+1|k+1}-N_{k|k+1}}{t_{cyc}}\begin{array}{cc} \Rightarrow & N_{k+1|k+1}=\frac{T-T_{L}}{J}t_{cyc}+N_{k|k+1} \end{array}$$

(12) $$t_{cyc}=\frac{1}{\frac{2N_{k|k+1}}{60}}=\frac{30}{N_{k|k+1}}$$

where, J is the rotational inertia; $T_{L}$ is the load torque; and $t_{cyc}$ is the working cycle time. T is the engine output torque and can be obtained by the empirical model [25], and its coefficients are obtained by calibrating the actual engine.

(13) $$\begin{array}{l} T=-181.3+379.36m_{air}+21.91R_{A/F}-0.85R_{A/F}^{2}+0.26\sigma +0.0028\sigma^{2}+0.027N_{k|k+1}-0.000107N_{k|k+1}^{2}+\ldots \\ 0.00048N_{k|k+1}\sigma +2.55\sigma m_{air}-0.05\sigma^{2}m_{air} \end{array}$$

where, $m_{air}$ is the mass of air in cylinder for combustion (g); σ is the spark advance angle (SAA).

In this case, only the load $T_{L}$ is variable, thereby satisfying Equation (14).

(14) $$T=C+DB\frac{1-\cos\theta }{N_{k|k}}+0.027N_{k|k+1}-0.000107N_{k|k+1}^{2}+0.00048N_{k|k+1}\sigma$$

where $C=-181.3+21.91R_{A/F}-0.85R_{A/F}^{2}+0.26\sigma +0.0028\sigma^{2}$, $D=379.36+2.55\sigma -0.05\sigma^{2}$.

From this analysis, the model for Part B can be described by:

(15) $$N_{k+1|k+1}=\frac{C+DB\frac{1-\cos\theta }{N_{k|k}}+0.027N_{k|k+1}-0.000107N_{k|k+1}^{2}+0.00048N_{k|k+1}\sigma -T_{L}}{J}\cdot \frac{30}{N_{k|k+1}}+N_{k|k+1}$$

By combining Equations (12) and (15), the speed iteration model can then be established:

(16) $$\begin{array}{l} N_{k+1|k+1}=\\ \frac{C+DB\frac{1-\cos\theta }{N_{k|k}}+0.027\sqrt{\frac{2\eta_{e}B\frac{\left(1-\cos\theta \right)}{N_{k|k}}}{J}}-0.000107\frac{2\eta_{e}B\frac{\left(1-\cos\theta \right)}{N_{k|k}}}{J}+0.00048\frac{2\eta_{e}B\frac{\left(1-\cos\theta \right)}{N_{k|k}}}{J}\sigma -T_{L}}{J}\cdot \frac{30}{\frac{2\eta_{e}B\frac{\left(1-\cos\theta \right)}{N_{k|k}}}{J}}+\frac{2\eta_{e}B\frac{\left(1-\cos\theta \right)}{N_{k|k}}}{J} \end{array}$$

Equation (16) can be expressed as

(17) $$N_{k+1|k+1}=g(N_{k|k},\theta )\Rightarrow N_{k+1}=g(N_{k},\theta )$$

where, g(⋅) is a nonlinear function.

The relationship among $N_{k|k}$, $N_{k|k+1}$, and $N_{k+1|k+1}$ is depicted in Figure 3.

### 2.2. Design of Extended Kalman Filter

Under actual working conditions, the throttle opening value can be decomposed into two parts: an ideal opening value and throttle position signal noise mainly due to EMI. In measuring the crankshaft speed process, speed data is affected by measurement noise from the crankshaft and process noise from the engine. Engine vibration caused by engine knock, etc., is transmitted to the crankshaft. In addition, the disturbance caused by road surface irregularity is also gradually transmitted to crankshaft. According to Kalman filter theory, these fluctuations are considered as process noise and need to be processed. These noise sources result in a huge accumulation error during pressure measurements, and the Kalman filter (KF) [29,30] is the most preferable filtering method for measurement and process noise. The KF is used to predict an optimal output for the current step based on the optimal output of the previous step and the observed value of the current step. However, for nonlinear systems, the KF cannot achieve optimal prediction. Therefore, the Extended Kalman filter (EKF) [31,32,33] is proposed by using the local linear property of nonlinear systems. State prediction is accomplished by using EKF gain to update state and error covariance. After linearization by computing the nonlinear function’s first order Taylor series and Jacobian matrix at the operating point, the EKF algorithm matches the KF and it comprises two parts: time update and state update. A discrete-time state-space model with a zero mean and a $Q_{k}$ variance in the white process noise $\left(w_{k-1}\right)$ is shown as

(18) $$X_{k}=g(X_{k-1})+w_{k-1}$$

where, $X_{k}$ is the state vector of the kth step, and g(⋅) is a nonlinear function.

The state observation can be written as

(19) $$Z_{k}=h(x_{k})+v_{k}$$

where $v_{k}$ is the white measurement noise with a zero mean; $R_{k}$ is the variance; and h(⋅) is a nonlinear function.

The prediction is shown below:

(20) $${\hat{X}}_{k|k-1}=g(X_{k-1})$$

The first-order Taylor series for g(⋅) at ${\hat{X}}_{k-1|k-1}$ and h(⋅) at ${\hat{X}}_{k|k-1}$ are obtained as shown in Equations (21) and (22), respectively.

(21) $$G_{k}={\frac{\partial g}{\partial X}|}_{{\hat{X}}_{k-1|k-1}}=g\left({\hat{X}}_{k-1|k-1}\right)+\frac{\delta g\left(X_{k-1}\right)}{\delta X_{k-1}}{|}_{X_{k-1}={\hat{X}}_{k-1|k-1}}\left(X_{k-1}-{\hat{X}}_{k-1|k-1}\right)$$

(22) $$H_{k}={\frac{\partial h}{\partial X}|}_{{\hat{X}}_{k|k-1}}=h\left({\hat{X}}_{k|k-1}\right)+\frac{\delta h\left(X_{k}\right)}{\delta X_{k}}{|}_{X_{k}={\hat{X}}_{k|k-1}}\left(X_{k}-{\hat{X}}_{k|k-1}\right)+v_{k}$$

Let:

(23) $$\Delta_{k}=X_{k}-{\hat{X}}_{k}$$

Defining $P_{k+1}$ as the covariance of $\Delta_{k+1}$, the transcendental error covariance can be calculated as

(24) $$P_{k+1|k}^{}=G_{k}P_{k}G_{k}^{T}+Q_{k}$$

The major purpose of the EKF is to obtain a minimum-variance state estimate. The gain matrix of the EKF is computed subject to minimization of the estimation error covariance.

(25) $$Tr\left[P_{k+1}^{x}\right]=\underset{K_{k+1}}{\min}!\Rightarrow K_{k+1}=P_{k+1|k}^{}H_{k}^{T}{\left(H_{k}^{T}P_{k+1|k}^{}H_{k}^{T}+R\right)}^{-1}$$

State prediction is accomplished by using EKF gain to update state and error covariance.

(26) $${\hat{X}}_{k+1}={\hat{X}}_{k+1|k}^{}+K_{k+1}(Z_{k}-H_{k}{\hat{X}}_{k+1|k}^{})$$

(27) $$P_{k+1}=(E-K_{k+1}H)P_{k+1|k}^{}$$

The observed vector is written as follows, where the observed matrix H=1:

(28) $$Z_{k}=H_{k}x_{k}+v_{k}$$

## 3. Calculation of Cylinder Pressure

In this paper, due to only related to the crankshaft speed and the amount of air and fuel injection in engine, this method can be applied to multi-cylinder internal combustion (IC) engines including four-cylinder engines. More specifically, many key parameters of ignition engine such as spark advance angle (SAA) need to be considered in the process of building virtual in-cylinder pressure sensor model. Accordingly, at present the application of the virtual sensor is only limited to spark ignition engines. In addition, now there are many ways to describe the status of engine working cycle such as Otto-cycle, Diesel-cycle, Sabtache-cycle, and Atkinson-cycle. Among the many cycles, the Otto-cycle optimally depicts the four-stroke ignition combustion engine. Taking Otto cycle of standard four-stroke engine as an example, but the virtual sensor method is not limited to the engine described by Otto cycle.

**Figure 4:**
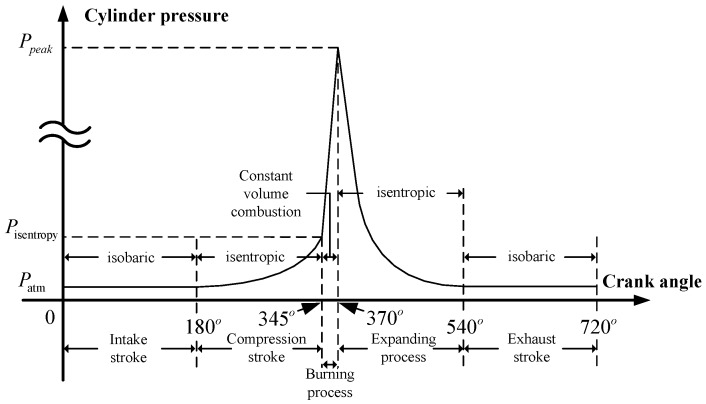
Diagram of the standard working cycle.

In this cycle, the crankshaft rotates 720° as one working period, where the 720° can be divided into four sub-cycles each with a crankshaft rotation of 180° [34]. A diagram of the standard working cycle is shown in Figure 4. These four smaller cycles are known as: intake stroke, compression stroke, power stroke, and exhaust stroke. It is the power stroke that involves the burning and expanding process. According to the Otto-cycle, the cylinder pressure during the intake stroke and the exhaust stroke is equal to atmosphere. The compression stroke and the expanding process are considered as isentropic, and the burning process is regarded as constant volume combustion [35].

The pressure at the end of compression stroke is also the initial pressure of the burning process. The peak occurs at the end of the burning process and is known as the peak pressure of the cylinder. This pressure is an important variate to understand the combustion efficiency of the burning process and to monitor the state of the cylinder.

### 3.1. Cylinder Pressure Peak Time Confirmation

It’s generally accepted that when the spark advance angle (SAA) is 10–15° after top-dead-center (TDC), combustion efficiency becomes maximum, while engine vibration reaches a minimum [35]. In order to obtain the maximum power of engine output at Optimal-Spark-Advance-Angle (OSAA), the OSAA need to be adjusted continuously.

In the case of high speed and low load of engine, the OSAA should be increased. This is due to the delay period of fuel combustion in the cylinder. The faster the speed is, the bigger the OSSA. On the contrary, with the speed increased, the stronger the turbulence formed by combustion gas in the cylinder is, the faster the combustion speed and the lower the advance angle is. combustion turbulence, which positively affects the burning speed, will increase with faster crank speeds. In this case, the corresponding OSAA delay period will be small and because of the delay caused by fuel burning in the power stroke, the OSAA should be slightly smaller. Additionally, in this paper, the pressure identification based on the power flow is adopted. The comparison between identified pressure signal and actual pressure signal can be realized by computing each power. Hence the SAA is deemed as constant. Due to the interaction and method selected, it’s deemed that the OSAA is constant at 15°, and the maximum power output will occur when the crankshaft angle is 10° after the TDC where pressure in the cylinder reaches its peak value.

### 3.2. Computation of Cylinder Pressure Peak Value

After the burning process, the exhaust gases in cylinders is primarily composed of $CO_{2}$, $H_{2}O$ and $N_{2}$.According to Equations (7) and (8), the specific heat capacity at constant volume for the burned gas is

(29) $$C_{mix}\approx (\frac{\frac{w_{C}}{12}}{\frac{w_{C}}{12}+\frac{w_{H}}{8}}C_{CO_{2}}+\frac{1}{2}\cdot \frac{\frac{w_{H}}{8}}{\frac{w_{C}}{12}+\frac{w_{H}}{8}}C_{H_{2}O})\cdot \frac{2}{9}+C_{N_{2}}\cdot \frac{7}{9}$$

where, $C_{CO_{2}}$ is the specific heat capacity at a constant volume for $CO_{2}$, and $C_{H_{2}O}$ is that for $H_{2}O$.

As computed in Equation (30), Cylinder temperature varies with output power.

(30) $$\Delta T=\frac{Q}{C_{mix}m}$$

where, m is the mass of the burned gas, and according to conservation of mass theory:

(31) $$m=m_{air}+m_{fuel}$$

According to the ideal gas state equation,

(32) pV=nRT

Since the burning process is deemed as isovolumetric, the peak pressure in cylinder is written as:

(33) $$p_{peak}=\frac{nR(\frac{Q}{C_{mix}(m_{air}+m_{fuel})}+T_{comp})}{V}$$

where, n is the amount of substance which can be gained from the coefficient of the fuel burning equation; V is the volume of cylinder at top dead center; $T_{comp}$ is the temperature at the end of the compression stroke; and R is the ideal gas constant.

## 4. Modeling of in-Cylinder Pressure

As previously mentioned, the cylinder pressure signal is periodic with variating frequency and amplitude, FAMFS is proposed to modulate the cylinder pressure [36]. A high-factorial Fourier series with variating frequency and amplitude is adopted to modulate the cylinder pressure.

### 4.1. Frequency-Modulated Fourier-Series

If there is a periodic signal f(x), then for every x there exists a positive value L which makes f(x)=f(x+L) correct, where L is known as the fundamental period and $\omega_{0}=\frac{2\pi }{L}$ is termed the fundamental frequency. The Fourier-series is written as [37]:

(34) $$f\left(x\right)=a_{0}+\sum_{n=1}^{\infty }\left(a_{n}\cos nx\frac{2\pi }{L}+b_{n}\sin nx\frac{2\pi }{L}\right)$$

where, $a_{0}$ is the intercept and $a_{n}$, $b_{n}$ are coefficients of the Fourier-series.

The frequency-modulated method [38] allows the instantaneous frequency of the carrier signal to vary with the change law of the delivery signal. This modulation can be classified as primary or secondary according to the category of the modulation effects.

With a defined carrier signal $x_{c}(t)=A\cos(2\pi f_{c}t)$ and a transmitted signal y(t), the modulating signal can be written as:

(35) $$x_{c}(t)=A\cos(2\pi \int_{0}^{t}\left[f_{c}+f_{\Delta }y(\tau )\right]d\tau )$$

where, $f_{c}$ is the center frequency of the carrier signal; A is the amplitude; $f_{c}+f_{\Delta }y(\tau )$ is the instantaneous frequency; and $f_{\Delta }$ is the frequency deviation gain.

Based on the engineering practice, the 24-order FAMFS is selected. It should be noted that the identification process is completed offline, and the only online requirement is adjustment of the frequency and amplitude of the FAMFS. Thus, the instantaneity of method is well guaranteed. The 24th order FAMFS is shown below:

(36) $$f\left(x\right)=Aa_{0}+A\sum_{n=1}^{24}\left(a_{n}\cos n(2\pi \sum_{t_{i}=0}^{t}\frac{t}{N_{n}}y(t_{i}))+b_{n}\sin n(2\pi \sum_{t_{i}=0}^{t}\frac{t}{N_{n}}y(t_{i}))\right)$$

where, t is the total running time; $N_{n}$ is the number of working cycles; and $t_{i}$ is time sequence.

### 4.2. Pressure Model

The optimal output of the Kalman observer is the speed of each power stroke. For a four-cylinder engine, the cylinders work in a logical sequence, known as firing order. Generally, the firing order is 1-3-4-2. By sampling the optimal output of the EKF, according to the working sequence, the optimal power-stroke speed for each cylinder is obtained:

(37) $$\begin{array}{l} N_{num1}=N_{1+kn}\\ N_{num2}=N_{2+kn}\\ \begin{array}{c} \cdot \\ \cdot \\ N_{numi}=N_{i+kn} \end{array} \end{array}$$

where, $N_{numi}$ is the speed of the ith cylinder; $N_{i+kn}$ is the optimal speed of (i+kn)th cycle; and k is a natural number.

According to actual working conditions, the cylinder pressure signal can be deemed as a FAMFS [39,40] signal with a center frequency at zero and a unit gain of frequency deviation. Thus, the pressure identification is ultimately written as:

(38) $$F(x)=A_{k}a_{0}+P_{atm}+A_{k}\sum_{n=1}^{24}\left(a_{n}\cos n(2\pi \sum_{t_{i}=0}^{t}\frac{t}{N_{n}}y(t_{i}))+b_{n}\sin n(2\pi \sum_{t_{i}=0}^{t}\frac{t}{N_{n}}y(t_{i})\right)$$

(39) $$y(t_{i})=\frac{\pi N_{k}}{15}\begin{array}{cc} & \sum_{n=1}^{k}\frac{2\pi }{\omega_{n}}\leq t_{i}\leq \sum_{n=1}^{k+1}\frac{2\pi }{\omega_{n}} \end{array}$$

(40) $$A_{k}=\frac{nR(\frac{Q_{k}}{C_{mix|k}(m_{air|k}+m_{fuel|k})}+T_{comp})}{V}\begin{array}{cc} & \sum_{n=1}^{k}\frac{2\pi }{\omega_{n}}\leq t_{i}\leq \sum_{n=1}^{k+1}\frac{2\pi }{\omega_{n}} \end{array}$$

where, t is time, and $A_{k}$ is the theoretical pressure peak at the kth working cycle.

## 5. Validation and Results

When the engine is in normal combustion state, its air-fuel ratio usually exceeds 0.85. A ratio of 0.7 or 0.8 will make the burning process of inside cylinder unstable and unpredictable, and this condition only happens in a very special condition like startup in an extremely cold environment or engine malfunctioned. No matter in which condition, it’s not a normal working status so the prediction is not that useful. The validity of the proposed method is verified by comparing the identification value of the virtual cylinder pressure sensor with the measured value through the cylinder pressure sensor.

In the following section, data collected from a genuine 2.0-L four-stroke four-cylinder engine was provided by the FAW Group Corporation R&D Center (Changchun, China). The engine model is EA211 and produced by VW (Volkswagen) (Changchun, China). Data was collected over the course of 100 working cycles adopting Kistler Model 6052A Pressure Sensor to test the performance of the proposed method.

### 5.1. Set Air-Fuel Ratio Coefficient at 0.85

**Figure 5:**
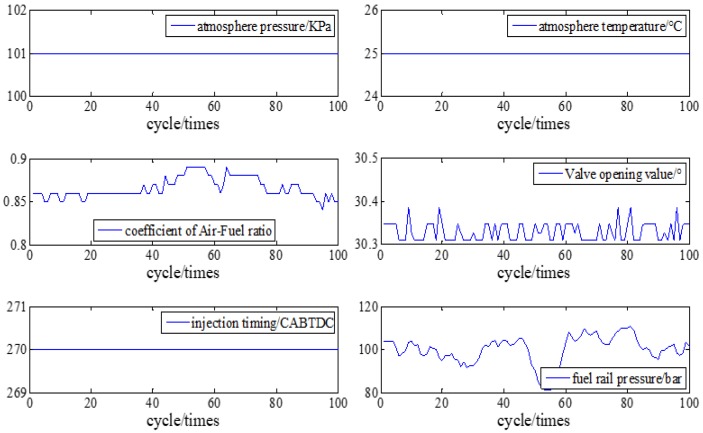
Parameters and setup of Engine at A/F ratio 0.85.

The parameters and setup of the engine are shown in Figure 5 and Table 1.

**Figure 6:**
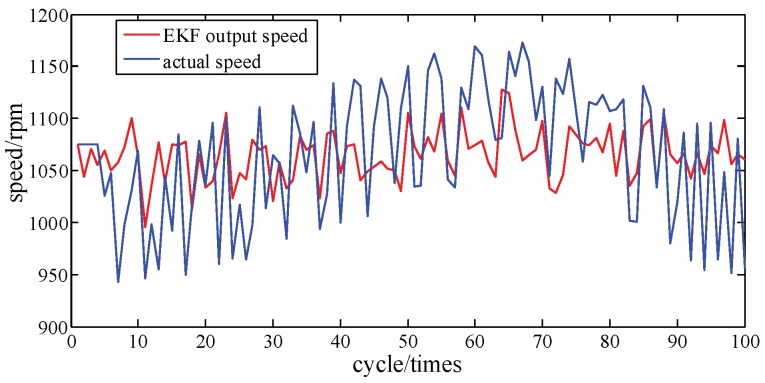
Speed tracking at A/F ratio 0.85.

**Figure 7:**
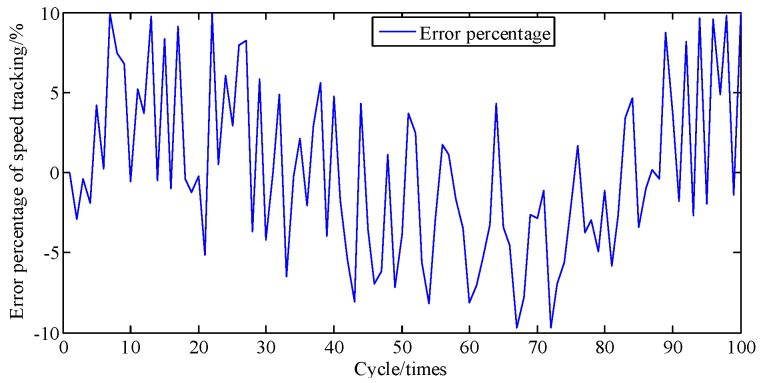
Error percentage of speed tracking at A/F ratio 0.85.

Figure 6 compares the crankshaft speed outputs of the EKF with the measured results acquired from the genuine engine. As seen in Figure 6 and Figure 7, from 30 cycles to 70 cycles the engine speed measured showed signs of rising and began to decline after reaching the maximum at 70 cycles. The EKF method accurately tracks this trend and its maximum percentage of error is only 9.6%. This shows acceptable agreement between identified and actual values.

**Figure 8:**
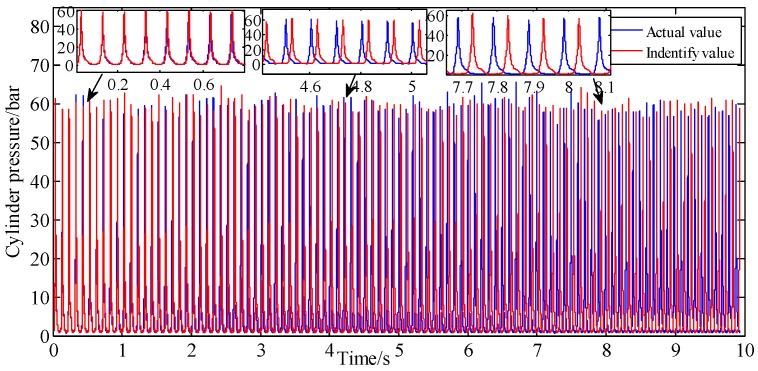
Pressure identification at A/F ratio 0.85.

Once the optimal speed from the EKF engine model is predicted, the peak pressure of cylinder can be calculated. Furthermore, the 24th order FAMFS is adopted for identification purposes, and its parameters are shown is Table 2:

A comparison of the actual cylinder pressure with the model-identified values is shown in Figure 8. It shows that in early stage of identification process, the identified values are basically in agreement with the measured values, whether in phase or amplitude. However, the phase deviation between measured and identified values begins to emerge and become larger with time. This is due to the cumulative error caused by the fitting error of the 24-order Fourier series and the speed tracking error.

**Figure 9:**
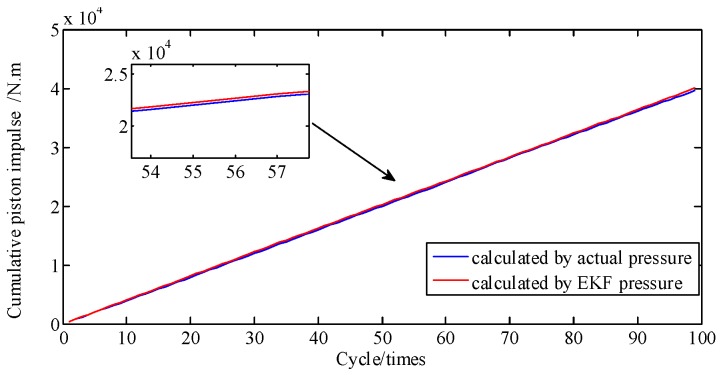
Piston impulse accumulated at A/F ratio 0.85.

**Figure 10:**
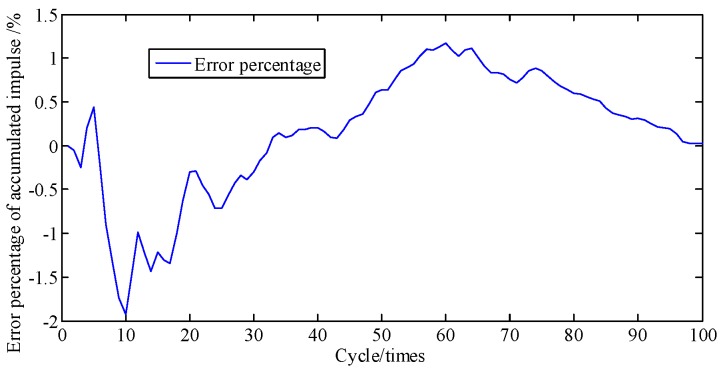
Error percentage of piston impulse accumulated.

For Figure 8, the area under the curve is multiplied by the piston area to represent the piston impulse. Hence, the cumulative error percentage of piston impulse is the cumulative error percentage of cylinder pressure. Figure 9 shows the curves of cumulative piston impulse including the actual and identification situation within 100 cycles. The two coincide basically. And Figure 10 shows that its cumulative error percentage is less than 1.8%. This agreement as well as the permitted errors, represents good performance from the proposed method.

**Figure 11:**
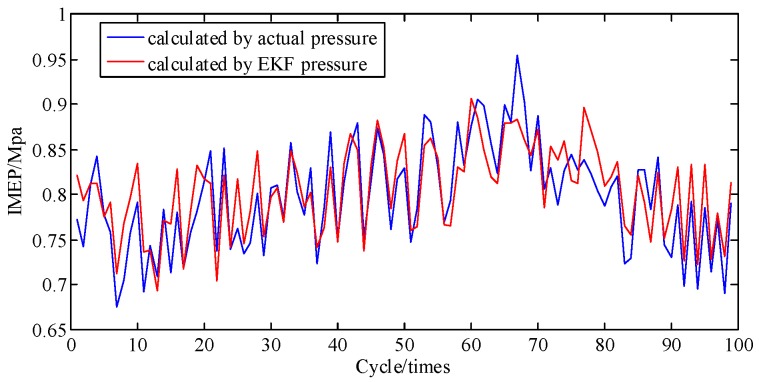
IMEP at A/F ratio 0.85 per cycle.

**Figure 12:**
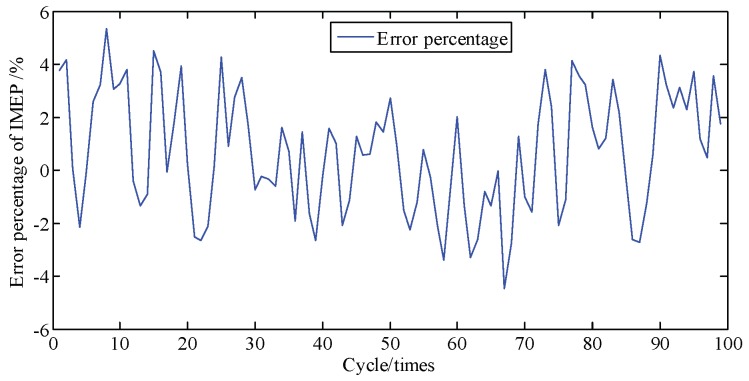
Error percentage of IMEP at A/F ratio 0.85 per cycle.

In general, the engine is a complex and sophisticated system, and small changes in valve or atmospheric temperature, fuel rail pressure, vibration from irregularity, etc., can severely impact the state of the engine; thus, besides a direct comparison of pressure and the cumulative piston impulse, comparison of piston impulse per cycle is also needed. As depicted in Figure 11, the actual and identified IMEP of each cycle one by one are contrasted. Figure 12 shows that these two error percentages are below 5.4%. And then the proposed method is proven to possesses a great ability to track the IMEP calculated by actual pressure.

### 5.2. Set Air-Fuel Ratio Coefficient at 0.95

**Figure 13:**
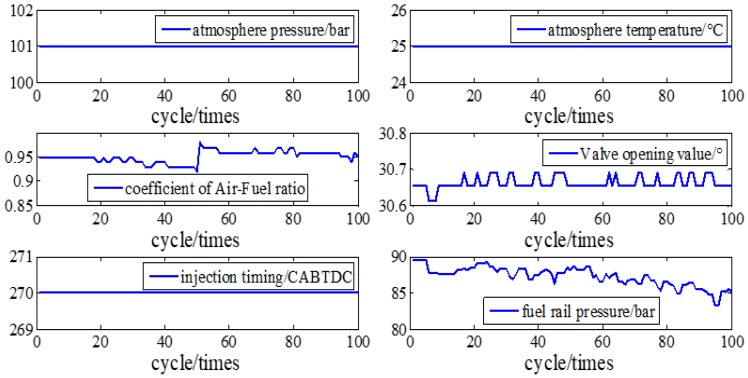
Parameters and setup of Engine at A/F ratio 0.95.

The parameters and setup of the engine are shown in Figure 13.

**Figure 14:**
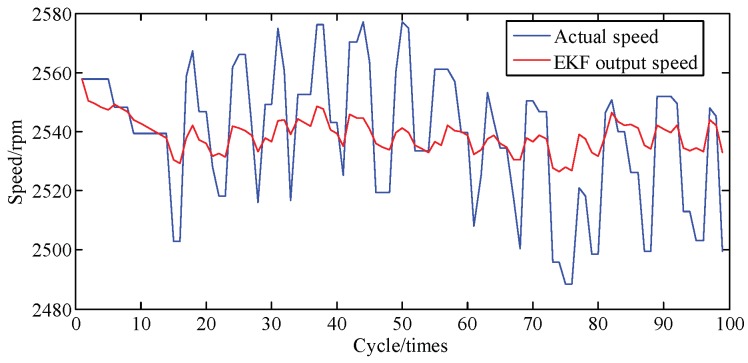
Speed tracking at A/F ratio 0.95.

**Figure 15:**
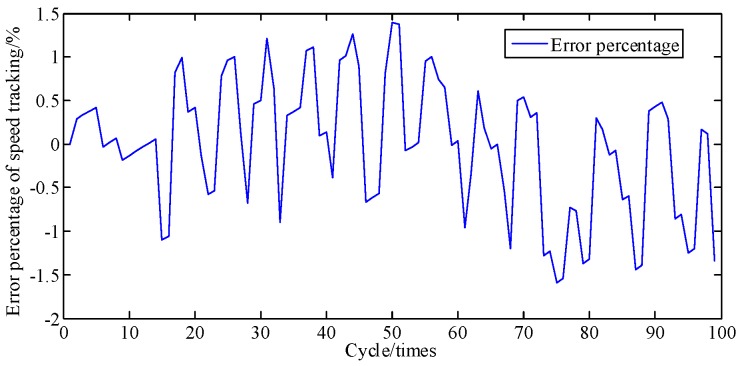
Error percentage of speed tracking at A/F ratio 0.95.

Figure 14 shows the comparing result of speed from EKF and a genuine engine at A/F ratio 0.95. Error percentages of the speed in Figure 15 is below 1.7%. Calculated and actual values have a great uniformity. Results show that proposed method possessed a great performance on tracking speed.

**Figure 16:**
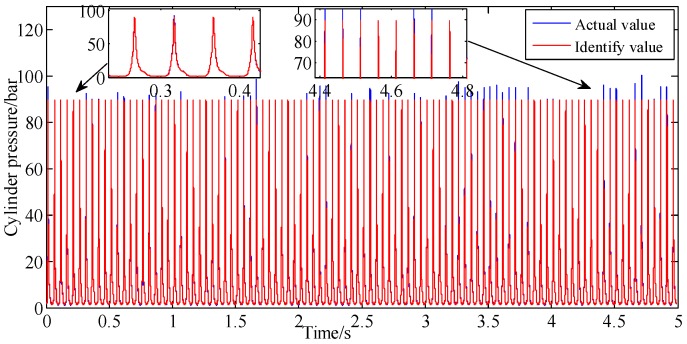
Pressure identification at A/F ratio 0.95.

The peak pressure of cylinder can be calculated by using the predicted speed. Furthermore, the 24th order FAMFS is adopted for identification purposes, and its parameters are the same as Table 2. A comparison of the actual cylinder pressure with the model-identified values is shown in Figure 16.

**Figure 17:**
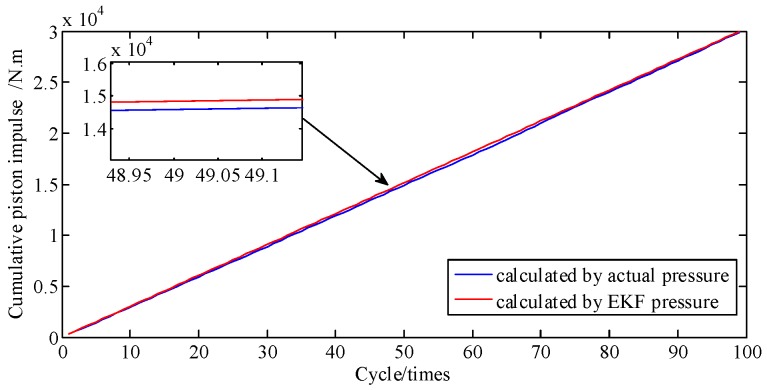
Piston impulse accumulated at A/F ratio 0.95.

**Figure 18:**
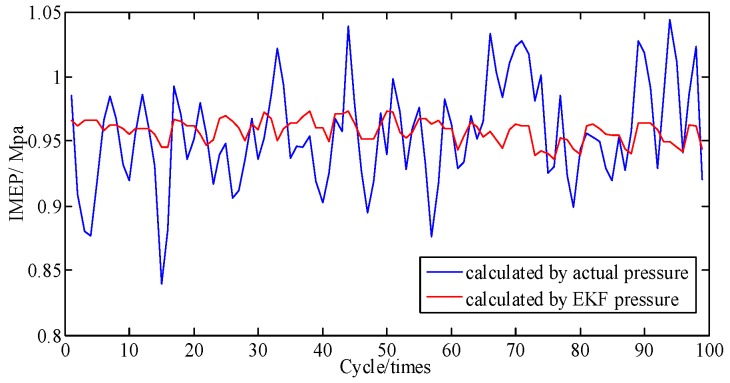
IMEP at A/F ratio 0.95 per cycle.

**Figure 19:**
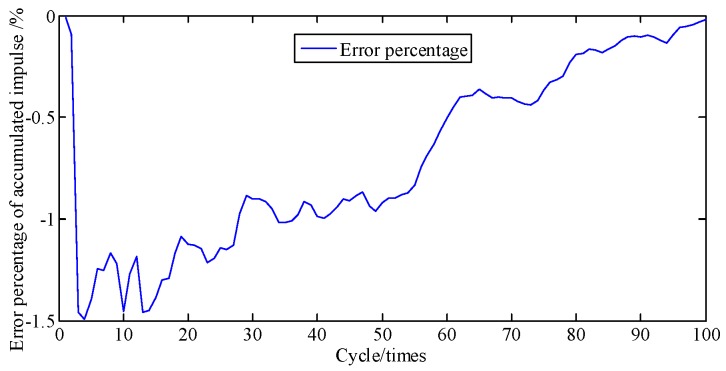
Error percentage of piston impulse accumulated.

**Figure 20:**
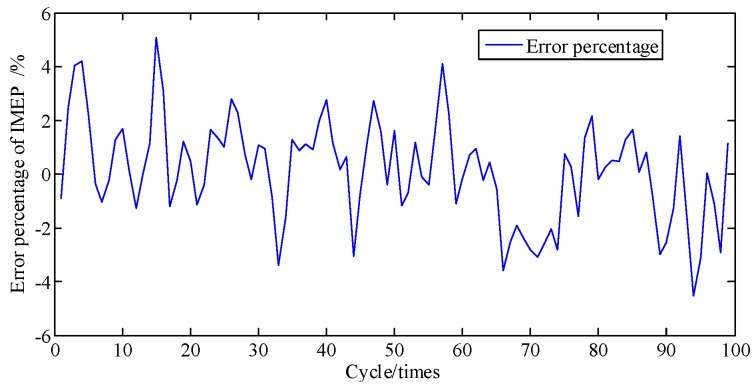
Error percentage of IMEP at A/F ratio 0.95 per cycle.

Figure 16 shows that the comparing result of actual and identified cylinder pressure. Figure 17 also shows the curves of cumulative piston impulse including the actual and identification situation but at A/F ratio 0.95 within 100 cycles. The two coincide also basically. As depicted in Figure 18, the actual and identified IMEP of each cycle one by one are contrasted. Compared with Figure 8 and Figure 16, the proposed method possesses a better ability to track the cylinder pressure at A/F ratio 0.95 than at A/F ratio 0.85. The closer the air-fuel ratio coefficient is to the optimal A/F ratio, the more predictable the combustion state of the engine is. This is also proved by the cumulative error percentage of piston impulse is less than 1.4% in Figure 19, and the error percentage of IMEP in each cycle is less than 4.9% in Figure 20. On the contrary, the phase difference of identified cylinder pressure began to emerge and become larger as the air-fuel ratio coefficient getting far away from optimal Air-Fuel ratio coefficient. But no matter how the A/F ratio coefficient changed, if it is within reasonable range, error percentage of cylinder pressure is within tolerance.

## 6. Conclusions

As a crucial and critical factor in monitoring the internal state of an engine, pressure identification is significantly essential. In this paper, aimed at solving problems associated with reliability and cost of invasive pressure sensors, virtual cylinder pressure identification sensor based on EKF and FAMFS is proposed. This new method employs three key steps:

(1) An iterative speed model based on burning theory and Law of energy Conservation. Efficiency coefficient is used to represent operating state of engine from fuel to motion. The iterative speed model associated with the throttle opening value and the crankshaft load.
(2) The EKF is used to estimate the optimal output of this iteration model. The optimal output of the speed iteration model is utilized to separately compute the frequency and amplitude of the cylinder pressure cycle-to-cycle.
(3) A pressure fitting algorithm is established by a 24th-order FAMFS. With this process an approximate identification method for engine cylinder pressure is developed in a standard engine’s working cycle.

To further verify the validity of the proposed method, data collected from a genuine engine (EA211) provided by the China FAW Group Corporate R&D Center was used. Test results demonstrate that the proposed method exhibits great performance for tracking crank speed and the engine’s real-time cylinder pressure. By comparing the identified pressure outputs with measurement results, the cumulative error percentage for cylinder pressure was below1.8%, the error percentage of IMEP each cycle was no more than 5.4%, and the error associated with speed was less than 9.6%, when A/F Ratio coefficient was set to 0.85. However, the cumulative error percentage for cylinder pressure was below 1.4%, the error percentage of IMEP each cycle was no more than 4.9%, and the error associated with speed was less than 1.7%, when A/F Ratio coefficient was setup at 0.95. The effectiveness and instantaneity are thereby proven and shown to be capable of achieving the desired accuracy despite the uncertainty of the engine’s power stroke. The closer the A/F ratio set to optimal A/F ratio, the more exact the identified pressure will be. Furthermore, it is important to note that the proposed method is valid, in general, and may be applied to more complex situations.

## Figures and Tables

**Table 1:** The parameters and setup of the engine.

A/F Ratio	Atmosphere Pressure/KPa	Atmosphere Temperature/°C	Valve Opening Value/°	CA BTDC	Fuel Rail Pressure /bar
0.85	101	25	30	270	100
0.95	101	25	30	270	90

**Table 2:** Parameters of the 24th order Fourier series.

a0~a6	a7~a13	a14~a20	a21~a24, b1~a3	b4~b10	b11~b17	b18~b24
12.06	−2.639	0.324	0.3783	5.68	−1.775	0.4077
−11.92	0.1302	0.7365	−0.3231	−5.136	0.9967	0.2995
−3.768	1.882	−0.9149	−0.0095	1.267	0.3929	−0.5088
9.395	−1.854	0.1323	0.257	1.927	−1.146	0.2242
−4.813	0.2843	0.5965	13.11	−2.787	0.7361	0.202
−1.215	1.142	−0.5427	−11.71	1.365	0.291	−0.3184
3.652	−1.264	−0.00471	1.303	0.8418	−0.8235	0.1462
